# Target margin design for real-time lung tumor tracking stereotactic body radiation therapy using CyberKnife Xsight Lung Tracking System

**DOI:** 10.1038/s41598-017-11128-w

**Published:** 2017-09-07

**Authors:** Zhi-Yong Yang, Yu Chang, Hong-Yuan Liu, Gang Liu, Qin Li

**Affiliations:** Cancer Center, Union Hospital, Tongji Medical College, Huazhong University of Science and Technology, Wuhan 430022, China

## Abstract

The objective of this study is to quantify the clinical accuracy of the Cyberknife Xsight Lung Tracking System (XLTS) in our center and calculate the PTV margin of XLTS treated lung tumors. Data from the treatment log files of 22 lung cancer patients treated with the CyberKnife XLTS were analyzed and the PTV margin was calculated. Segmentation, deformation, correlation, prediction and targeting errors were calculated from the log files of XLTS treatments. Two different methods were used to calculate anisotropic treatment margin. The relationships between tumor motion ranges and the correlation and prediction errors were also analyzed. Based on our estimation of a 4 mm global margin, 95% coverage in the S-I direction and 100% coverage in the L-R and A-P directions were obtained. Strong correlations between tumor motion range and the standard deviation (SD) of correlation and prediction errors were also found. Tumor position motion caused by respiration can be compensated using the Xsight Lung Tracking System. We found total tracking errors to be less than 4 mm in all three directions. This result could provide a reference for the selection of PTV margin for treatment with the CyberKnife XLTS.

## Introduction

The tumor motion induced by respiration is particularly challenging when delivering high dose hypo-fractionated treatments to lung cancer patients, such as stereotactic body radiation therapy (SBRT). In the absence of real-time tumor tracking and adjustments, an internal margin is added to encompass the entire range of target motion and rotations induced by respiration, and an internal target volume (ITV)-to-planning target volume (PTV) margin is also needed to compensate for the geometrical uncertainty from localization, set-up, delivery and other sources^1, 2^. However, the margin can significantly increase the treatment volume and include the surrounding tissues^3, 4^, and it could increase the lung toxicity sometimes^5^. Furthermore, the ITV standard is limited by the uncertainties inherent to respiratory motion, which is not purely periodic^6^. Hence, real-time image-guided treatment is needed to reduce the internal margin and improve the delivery accuracy of SBRT.

The CyberKnife robotic radiosurgery system (Accuray, Inc., Sunnyvale, CA, USA) is an option that is able to provide high accuracy and sub-centimeter margin. The CyberKnife has two respiratory tracking systems: the fiducial-based target tracking system (FTTS) and the Xsight Lung Tracking System (XLTS)^7^. The XLTS is a fiducial-free real-time tracking system used to irradiate lung tumors that moves with respiration^8^. The advantage of the XLTS is that it is non-invasive, thereby eliminating the risk of fiducial insertion-related adverse events, such as pneumothorax or pulmonary hemorrhage^9–11^. Patients can breathe normally throughout XLTS treatment while the CyberKnife robot arm actively compensates for tumor motion. For those large lesions (diameter >15 mm) located in the peripheral regions of the lung, the density difference between the lung and the solid tumor is sufficient to extract the position of the target from the X-ray images. The XLTS computes the discrepancy between the real-time images of the tumor and the correlation model, and then the CyberKnife robot arm automatically adjusts for the discrepancy of the tumor positions.

Due to the tumor motion baseline and trajectory changes, set-up errors and anatomical changes between treatment fractions, the tumor position needs to be reconfirmed on each fraction before treatment^12^. The inter-fraction treatment errors can be reduced by the image-guided radiotherapy^12^. However, tumor motion during each fraction can lead to a high intra-fraction error and a larger margin expansion. The intra-fraction tumor motion induced deviation of planned dose and actual delivered dose depends linearly on the dose rate gradient and the range of motion. At the same time, dose deviation is inversely proportional to the frequency of tumor motion. Therefore, a lower dose rate gradient, a smaller tumor motion range or a higher respiratory frequency can reduce the deviation^13^.

There are some studies regarding the tracking errors of the CyberKnife respiratory tracking system (FTTS and XLTS). The tracking errors of FTTS and XLTS has similar multiple sources: the geometric or targeting error of the CyberKnife radiation delivery system; the correlation error between internal tumor locations versus external respiratory surrogate positions; the prediction error for system delay compensation; the tumor segmentation error and the tumor deformation error. The deformation error comes from the inner deformation of the tumor during treatment^14^. However, there are certain differences between the tumor segmentation errors of the two systems. In the FTTS, the tumor segmentation error comes from the differences in the distance between the center of mass (CoM) of fiducials in the digital reconstructed radiographs (DRRs) created by the planning CT and the CoM of the fiducials in the real-time X-ray images, which may deform during treatment. In the XLTS, the tumor segmentation error is the relative position of the CoM of the tumor in DRRs and the CoM of the tracking target in the real-time X-ray images. The segmentation error of the XLTS depends on tumor density, soft-tissue contrast and the segmentation ability of X-ray images. However, sufficient tumor contrast against the surrounding tissues in X-ray images is essential for reducing the tumor segmentation error of the XLTS. Several studies have investigated the tracking accuracy of the FTTS based on clinical log file analysis or phantom study^15–19^, but there is seldom research about the tracking error and margin analysis of the XLTS. Descovich *et al*. analyzed real-time respiratory tracking system clinical data including both FTTS and XLTS, and determined the ITV-to-PTV margin^20^. However, the tracking error of the XLTS was not reported. Jung *et al*. have analyzed the segmentation error of the FTTS and XLTS using lung phantoms created with precision 3D-printing technology, and in both systems they found, the segmentation errors to be less than 1.5 mm, which means that the two systems have comparable segmentation accuracy^21^. Fu *et al*. evaluated the distance between tumor centroids on X-ray images and the planning CT, and the tracking error (including the tumor segmentation error) of the X-ray images was 1.06 ± 0.19 mm^8^. This value is much smaller than FTTS results reported before^15–17^, and does not agree with the hypothesis that the tracking error of the XLTS is greater than that of the FTTS. The total tracking error of the XLTS in clinical treatment is still unclear, and the individual margin of the lung tumor treated by the XLTS has not been reported.

In this study we evaluated the relationship between the tumor positions and tracking errors, calculated the margin using a previously validated formulation^17, 20^ and the uncertainty estimation^14, 15, 22^. The margin was calculated from whole fractions data, in order to analyze the un-periodic respiratory motion during the treatments.

## Materials and Methods

### Patient characteristics

Twenty-two lung tumor patients treated for a total of ninety-two fractions were analyzed (with 3–5 fractions for each patient). All patients were treated on the CyberKnife VSI system using 2-view XLTS. This study was approved by the Institutional Review Board at the Tongji Medical College of Huazhong University of Science and Technology. All methods were carried out in accordance with the relevant guidelines and regulations. Written informed consent was obtained from all subjects.

Patient characteristics are presented in Table 1, categorized by anatomical location (upper, middle, and lower lobe) and degree of centrality (central vs peripheral). Central lesions are located within or touching the zone of the proximal bronchial tree^23^, defined as a volume 2 cm in all directions around the proximal bronchial tree, while lesions outside this area are defined as peripheral. Tumor volume, density and tumor motion ranges over the respiratory cycle are also reported in Table 1. Tumor motion range is 1–99% of the range of target motion positions.

**Table 1:** Patient and target motion characteristics (Patient number = 22; Treatment fractions = 92).

Characteristics	
**Anatomical location:**	
Upper lobe	9
Middle lobe	7
Lower lobe	6
**Tumor location**	
Peripheral	9
Central	13
Fractions: Mean ± SD	4.2 ± 2.1
Treatment time: Mean ± SD/median (minute)	28.6 ± 7.4/26.0
Tumor density: Mean ± SD/median (g/cm^3^)	0.88 ± 0.08/0.85
Tumor volume: Mean ± SD/median (cm^3^)	12.7 ± 7.8/7.5
Tumor size (the longest lengths in three dimensions): Mean (cm)	3.0 * 2.2 * 2.0
Tumor motion range*: Mean ± SD (mm)	
S-I direction	9.0 ± 7.3
L-R direction	8.5 ± 5.6
A-P direction	4.8 ± 3.5

*Tumor range is the 1–99% range of target positions in each direction obtained from each treatment fraction.

Patients were immobilized with vacuum pads in supine position, with their arms along their sides. First, the XSight lung tumor visualization test was carried out to confirm that the tumor is seen clearly on both X-ray detectors. Treatment planning was performed with XLTS procedures. The gross tumor volume (GTV) was contoured on the exhale phase CT images and used for target tracking by the XLTS. The GTV to clinical target volume (CTV) margin for inclusion of microscopic extension of the tumor was 2 mm. Depending on the specific clinical scenario and previous FTTS experience, the PTV is derived using a 5 mm expansion from the CTV in all three directions to account for treatment uncertainties and residual errors in our center^15–17^.

### Treatment delivery

During the treatment, the patients wore a tightly fitting vest with three infrared light emitting diodes (LEDs) on the anterior of the vest. Tumor position was provided by the CoM of the tumor, which was automatically extracted from the X-ray images and back-projected to reconstruct the 3D coordinates in the patient’s coordinate system. The time interval between two adjacent X-ray imaging sessions was 40 seconds.

### Data collection

During each XLTS treatment, the CyberKnife generated several log files, of which the following 4 files were used for off line analysis:

1. The ModelPoints.log file, which recorded the estimated tumor position $({X}_{i},{Y}_{i},{Z}_{i},{T}_{i})$, at the time of X-ray imaging ${T}_{i}(i=1,2,3\cdots )$, at about 40 second intervals. Approximately 30–50 image pairs were taken for each XLTS treatment.
2. The Modeler.log file, which recorded the tumor positions $({x}_{j},{y}_{j},{z}_{j},{t}_{j})$, ${t}_{j}(j=1,2,3\cdots )$ estimated from the external LED markers through the correlation model at the rate of 26 Hz.
3. The Predictor.log file, which contained the output of the prediction model (predictor points) regarding the tumor positions $({x}_{j}^{^{\prime} },{y}_{j}^{^{\prime} },{z}_{j}^{^{\prime} },{t}_{j}^{^{\prime} })$, ${t}_{j}^{^{\prime} }(j=1,2,3\cdots )$ at 26 Hz. The tumor positions were predicted 115 milliseconds (ms) in advance, giving the robot arm time to correct for motion.
4. The ERsiData.log file, which recorded time-stamped information about the location of the robot and its adjustment for tumor tracking. This file was used to identify the times when radiation was being delivered. Only data that matched in time with the dose delivery of a treatment beam was used for analysis.

The 3D coordinates of the tumor positions in the ModelPoints.log recorded in the patient coordinate system and the Modeler.log files are recorded in recorded in the robot coordinate system^20^. A rotation matrix is used to transform all data from the robot coordinate system into the patient coordinate system.

The probability distribution functions (PDF) of tumor positions were calculated using the data in the Modeler.log file. The tumor motion range is 1–99% of the tumor motion positions $({x}_{j},{y}_{j},{z}_{j},{t}_{j})$, ${t}_{j}(j=1,2,3\cdots )$ in one treatment fraction.

### Data analysis

#### Estimation error sources

Since the CyberKnife SBRT is real-time image guided, the inter-fraction setup error is reduced, and the main error is intra-fraction error caused by respiration-induced target motion. In order to determine arbitrary points inside the CTV, positioned in relation to the delivered beams, the process can be split into five independent steps, representing the main intra-fraction error sources for margin expansion:

Step 1. The segmentation error, which is the discrepancy between the relative positions of the tracked target’s CoM of in DRRs and real-time X-ray images.

Step 2. The deformation error, which is the discrepancy between the relative positions of arbitrary points in target and the target’s CoM in real-time X-ray images and planning CT images.

Step 3. The correlation error, which is the discrepancy between the position of the correlation model and the position indicated in the X-ray images.

Step 4. The prediction error, which is the discrepancy between the position of the prediction model and the position of the correlation model 115ms in the future.

Step 5. The targeting error of the CyberKnife, which is the discrepancy between the intended target position and where the CyberKnife actually delivers radiation (including the robotic manipulator precision and radiation field accuracy).

Since the segmentation error depends on tumor density, soft-tissue contrast, and the segmentation ability of X-ray images, it is difficult to be distinguished and calculated retrospectively. We derived the error based on the study of Jung *et al*.^21^, who used patient-specific lung phantoms to evaluate the segmentation error of the XLTS. The total tracking error was 0.38 ± 0.54 mm, 0.13 ± 0.18 mm, and 0.14 ± 0.37 mm for the S-I, L-R, and A-P directions, respectively. Since Jung *et al*. minimized the correlation error and the prediction error by fitting the phantom movement using linear fit, the total tracking error of the XLTS, calculated by determining the 3D distance between the predicted tumor position and the actual target position in X-ray images, could partly represent the segmentation error of the XLTS. Due to the limitation of their study (the patient-specific lung phantoms were moving only in the S-I direction), we assigned the segmentation error to be 0.38 ± 0.54 mm in all three directions. For each direction *i* and patient *j* the uncertainty was calculated as ${U}_{S}^{ij}={\mu }_{S}^{ij}+2{\sigma }_{S}^{ij}$, where *μS* is the absolute mean, and *σ*_*S*_ is the standard deviation from the segmentation error distribution of whole treatment fractions.

The deformation error is dependent on individual patient features (e.g., inner tumor deformation, tumor motion range and speed), being one of the main sources of uncertainty. We assigned this error based on the studies of Smith *et al*.^24^ and Lu *et al*.^25^. Smith *et al*. studied the deformation error in lung tumors and plotted the deformation error as a function of the distance from the tumor centroid (Figure 5 in Smith *et al*.^24^). According to this study, the motion deformation error increases as the distance from the tumor centroid increases. Lu *et al*. find that a 3 mm margin could cover the deformation error and keep the dose coverage of CTV above 95%. Based on these information and depending on tumor size and the motion range of the tracked target’s CoM, we defined the deformation error as follows: 1) if the motion range of the tracked target’s CoM is not larger than 2 cm or it is inside the CTV, the deformation error and uncertainty is 1.5 mm in all three directions; 2) if the motion range of the tracked target’s CoM is larger than 2 cm or it is outside the CTV, the deformation error and uncertainty is 2.5 mm in all three directions. This error simultaneously takes into account deformations and rotations of the tumor inside the CTV and variations in the movement of any CTV point during the treatment. As all patients presented one of these two options, no extra conditions were added.

In each fraction, the correlation tumor positions $({x}_{i},{y}_{i},{z}_{i},{T}_{i})$ from the ModelPoints.log file corresponding to the time ${T}_{i}(i=1,2,3\cdots )$ of X-ray imaging were interpolated and then the shift of each data pair was calculated as:

1 $$Cerror({e}_{i}^{x},{e}_{i}^{y},{e}_{i}^{z},{T}_{i})=({X}_{i}-{x}_{i},{Y}_{i}-{y}_{i},{Z}_{i}-{z}_{i},{T}_{i}),{T}_{i}(i=1,2,3\cdots )$$

For each direction *i*, and patient *j*, the uncertainty was calculated as:

2 $${U}_{C}^{ij}={\mu }_{C}^{ij}+2{\sigma }_{C}^{ij}$$

where *μ*_*C*_ is the absolute mean, and ﻿*σ﻿*_*C*_ is the standard deviation (SD) from the correlation error distribution in whole treatment fractions.

In each fraction, the prediction error was derived by comparing the output tumor position of the prediction model with the correlation model 115 ms in the future. For each data pair of Modeler.log file and predictor.log file points, the shift was calculated as:

3 $$Perror({e}_{j}^{x},{e}_{j}^{y},{e}_{j}^{z},{t}_{j})=({x}_{j}-{x}_{j}^{^{\prime} },{y}_{j}-{y}_{j}^{^{\prime} },{z}_{j}-{z}_{j}^{^{\prime} },{t}_{j}),{t}_{j}(j=1,2,3\cdots )$$

For each direction *i*, and patient *j*, the uncertainty was calculated as:

4 $${U}_{P}^{ij}={\mu }_{P}^{ij}+2{\sigma }_{P}^{ij}$$

where *μ*_*P*_ is the absolute mean, and *σ*_*P*_﻿ is the SD from the prediction error distribution in whole treatment fractions.

The targeting error of the CyberKnife was measured in our center based on Xsight Lung phantom end-to-end (E2E) tests conducted before each patient’s XLTS treatments. The targeting error of E2E tests was recorded and calculated for in all three directions. For all patients, the uncertainty was calculated as:

5 $${U}_{T}^{i}={\mu }_{T}^{i}+2{\sigma }_{T}^{i}$$

For the direction *i*, *μ*_*T*_ is the absolute mean, and *σ*﻿_*T﻿*_ is the SD from the prediction error distribution in whole treatment fractions.

### Individual margin calculation

Two different methods were used to calculate the patient individual anisotropic margin.

**1. Using previously validated formulation** ^17, 20^ **:**

Van Herk *et al*. have described systematic error as error mainly due to the preparation of the treatment and random error as error during the delivery of the treatment every fraction^26^. The individual systematic error, ${\rm{\Sigma }}$, is the standard deviation SD of the means and is an assessment of reproducibility of the treatment preparation. And individual random error, $\sigma$, is the root mean square of the SD of the daily measurements^26, 27^. In this study, segmentation, deformation, and targeting errors are systematic error from treatment preparation. The correlation error and the prediction error were from execution variations, and both have systematic and random contributions.

The root-mean-square of the individual random error $\sigma$ was calculated as^20^:

6 $${\sigma }_{f}=\sqrt{\frac{1}{{N}_{mf}-1}\sum _{m=1}^{{N}_{mf}}{({e}_{mf}-{\langle e\rangle }_{f})}^{2}},$$

and

7 $$\sigma =\sqrt{\frac{1}{{N}_{f}}\sum _{f=1}^{{N}_{f}}{({\sigma }_{f})}^{2}}.$$

where ${e}_{mf}$ is the $m$ th shift observed for fraction $f$, ${\langle e\rangle }_{f}$ is the mean shift for fraction $f$(the mean of correlation/prediction error for fraction $f$), ${N}_{mf}$ is the number of data points in fraction $f$, ${\sigma }_{f}$ is the SD of correlation/prediction error for fraction $f$, and ${N}_{f}$ is the total number of treatment fractions. The SD of the individual systematic error ∑ was calculated as^20^:

8 $${\rm{\Sigma }}=\sqrt{\frac{1}{{N}_{f}-1}\sum _{f=1}^{{N}_{f}}{({\langle e\rangle }_{f})}^{2}}.$$

The anisotropic margin was calculated according to a previously validated formulation^17, 20^. In the formulation of van Herk *et al*., the margin (M) was calculated as:

9 $$M=2.5{\rm{\Sigma }}+\beta \sqrt{{\sigma }^{2}+{\sigma }_{p}^{2}}-\beta {\sigma }_{p}=2.5\sqrt{{{\rm{\Sigma }}}_{S}^{2}+{{\rm{\Sigma }}}_{D}^{2}+{{\rm{\Sigma }}}_{C}^{2}+{{\rm{\Sigma }}}_{P}^{2}+{{\rm{\Sigma }}}_{T}^{2}}+\beta \sqrt{{\sigma }_{C}^{2}+{\sigma }_{P}^{2}+{\sigma }_{p}^{2}}-\beta {\sigma }_{p}.$$

where ∑_*S*_ is the SD of the segmentation error, ∑_*D*_ is the SD of the deformation error, ∑_*T*_ is the SD of the targeting error, ${\sigma }_{p}$ represents the penumbra width of the radiation beam and $\beta$ depends on the dose level selected for dose prescription. In lung SBRT the width of the penumbra is broader than in water and due to the steep dose fall-off, a dose level of 80% might be applied to the prescription. In this situation, the values ${\sigma }_{p}$ = 6.4 mm and $\beta$ = 0.84 were chosen for calculation^26^. The margin formulation assumes 95% isodose coverage, and a 90% probability level (90% of the patient population).

2. **Using the uncertainty estimation method** ^14, 15, 20, 22^:

We tried to establish a combined expanded uncertainty for the whole treatment, and each individual uncertainty was assigned to each step. We evaluated all the error sources independently: all of them were considered Type B uncertainties. Because, in some cases, we used parameters that were only indirectly associated with the uncertainties of the studied steps or we were not able to obtain statistical information directly. In such cases, uncertainties were assigned based on the available literature^14, 21, 24^. When simplifications were made, we were always conservative in our analysis in order not to underestimate the final combined uncertainty of the process. Following the calculation steps shown in Table 2, we were able to estimate the expanded uncertainty (k = 2) for the whole treatment process of 95% coverage of the CTV points, with a 95% confidence level. Based on geometrical considerations only, we believe this combined uncertainty is an estimation of the individual PTV margin.

**Table 2:** The calculation steps of combined uncertainty of XLTS.

	${U}_{S}$	${U}_{D}$	${U}_{C}$	${U}_{P}$	${U}_{T}$
Uncertainty estimated type B	Segmentation uncertainty	Deformation uncertainty	Correlation uncertainty	Prediction uncertainty	Targeting uncertainty
Standard uncertainty (assuming normal pdf)	*SU*_*Segmenation*_ = *U*_*S*_/2	*SU*_*Deformation*_ = *U*_*D*_/2	*SU*_*Correlation*_ = *U*_*C*_/2	*SU*_*Prediction*_ = *U*_*P*_/2	*SU*_*Targeting*_ = *U*_*T*_/2
Combined standard uncertainty	$S{U}_{Combined}=\sqrt{S{U}_{Segmentation}^{2}+S{U}_{Deformation}^{2}+S{U}_{Correlation}^{2}+S{U}_{Prediction}^{2}+S{U}_{Targeting}^{2}}$				
Expanded uncertainty (k = 2, assuming normal pdf)	${U}_{Combined}=2\cdot S{U}_{Combined}=\sqrt{{U}_{S}^{2}+{U}_{D}^{2}+{U}_{C}^{2}+{U}_{P}^{2}+{U}_{T}^{2}}$				

### Statistical analysis

The target margin of the CyberKnife XLTS was calculated using the method presented above. Pearson correlation analyzes was used to test the relationship between the SD of correlation/prediction error and the tumor motion ranges of 92 treatment fractions, as well as between the uncertainty of correlation/prediction errors and the tumor motion ranges of 22 patients. The independent t-test were applied to compare the margin and uncertainty of XLTS in all three directions between the lesions located in upper/middle and lower lobe, peripheral lesions and central lesions, larger lesions and small lesions (tumor sizes above mean value vs. tumor sizes below mean value), larger motions and small motions (tumor motion ranges above mean value vs. tumor motion ranges below mean value). A P-value of 0.05 or less was defined as statistically significant.

## Results

### Error sources

Step 1: The segmentation errors were 0.38 ± 0.54 mm in all three directions based on the definition and previous data^21^. The SD of the segmentation error (∑_*S*_) was 0.54 mm in all three directions, and the uncertainly of segmentation error (${U}_{S}$) was 1.46 mm in all three directions.

Step 2: The deformation errors were 1.5 mm for most patients, and only two patients had a deformation of 2.5mm. According to the real-time Xsight Lung tracking and adjusting during the whole treatments, the mean of deformation error in each fraction is expected to be zero in each direction^26, 27^, we assigned the SD of the deformation error (∑_*D*_) to be 0.75/1.25 mm, and the uncertainly of the deformation error (${U}_{D}$) was 1.5/2.5 mm in all three directions.

**Figure 1:**
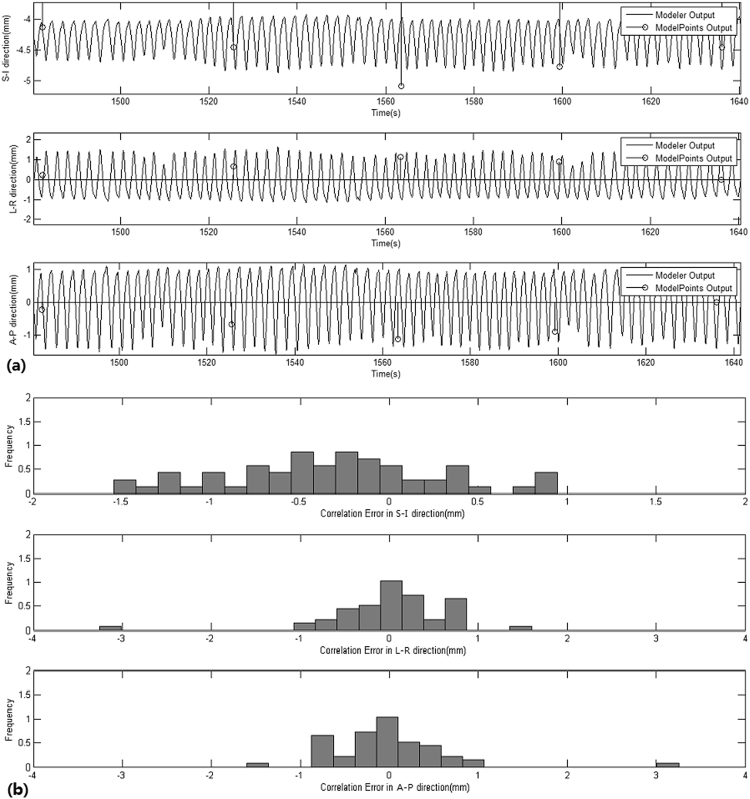
The correlation error (**a**) and frequency of correlation error (**b**) of one fraction﻿ ﻿is shown with the tumor position output of correlation model (solid line), the tumor position of X-ray imaging from the ModelPoints.log file (stem points) in three directions, respectively. The time intervals between the X-ray imaging is about 40 seconds.

**Figure 2:**
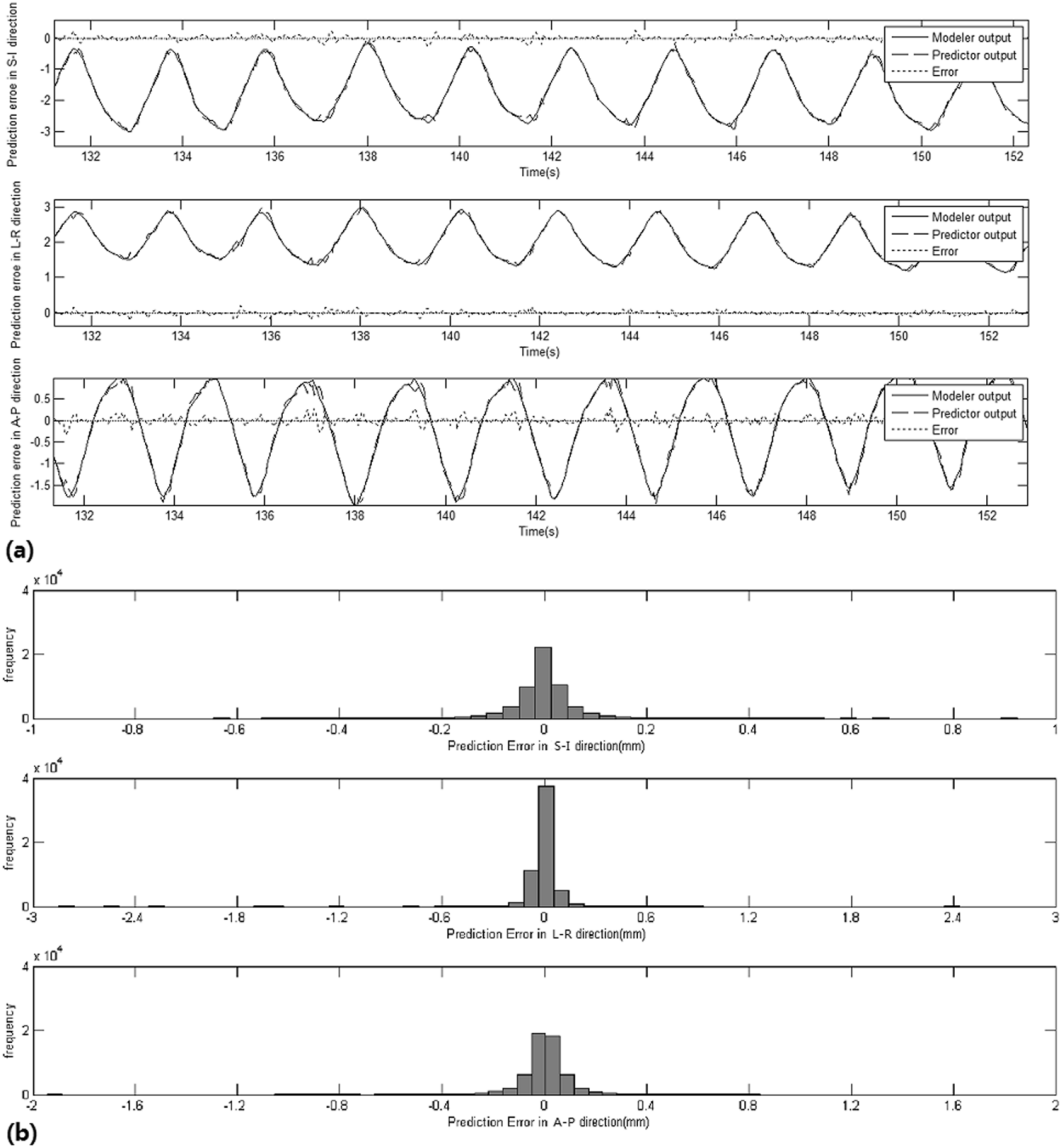
The prediction error (**a**) and frequency of prediction error (**b**) of one fraction with the tumor position output of correlation model (solid line), the tumor position output of prediction model (dashed line), and error of prediction (dotted line) in three directions, respectively. As the tumor position range, period changes, the prediction error increases. The prediction error is higher at the end of inspiration in the period of respiratory.

Step 3 and 4: The correlation error and the prediction error were calculated from each patient’s specific data. The systematic and random of the correlation error and the prediction error were calculated using equations (7) and (8). The uncertainties of the correlation error and the prediction error were calculated using equations (2) and (4). The correlation error was less than 1 mm in all cases, and the prediction error was much too small to be distinguished. All results are shown in Tables 3 and 4. The correlation error and the prediction error results of a sample treatment are shown in Figs 1(a,b) and 2(c,d).

**Table 3:** The values of segmentation error, deformation error, correlation error and prediction error and targeting error for each patient.

Patient	∑_*S*_	∑_*D*_	∑_C_/*σ*_c_ (mm)			∑_*P*_/*σ*_*P*_ (mm)			∑_*T*_ (mm)		
number	(mm)	(mm)	S-I	L-R	A-P	S-I	L-R	A-P	S-I	L-R	A-P
1	0.54	0.75	0.4/0.6	0.3/1.0	0.1/1.0	0.0/0.0	0.0/0.1	0.0/0.1	0.18	0.33	0.25
2	0.54	0.75	0.1/0.8	0.4/1.3	0.2/1.1	0.0/0.2	0.0/0.2	0.0/0.1	0.18	0.33	0.25
3	0.54	0.75	0.3/1.5	0.4/1.6	0.4/1.6	0.0/0.1	0.0/0.1	0.0/0.1	0.18	0.33	0.25
4	0.54	0.75	0.3/1.0	0.3/1.3	0.3/1.3	0.0/0.1	0.0/0.2	0.0/0.2	0.18	0.33	0.25
5	0.54	1.25	0.3/0.9	0.1/0.5	0.1/0.5	0.0/0.2	0.0/0.2	0.0/0.0	0.18	0.33	0.25
6	0.54	1.25	0.2/1.0	0.1/0.6	0.3/1.6	0.0/0.1	0.0/0.1	0.0/0.1	0.18	0.33	0.25
7	0.54	0.75	0.2/0.9	0.1/0.6	0.1/0.7	0.0/0.1	0.0/0.1	0.0/0.0	0.18	0.33	0.25
8	0.54	0.75	1.4/2.2	0.2/0.4	0.1/0.5	0.0/0.3	0.0/0.3	0.0/0.1	0.18	0.33	0.25
9	0.54	0.75	0.2/1.0	0.1/0.3	0.1/0.3	0.0/0.2	0.0/0.1	0.0/0.0	0.18	0.33	0.25
10	0.54	0.75	0.7/1.6	0.2/1.8	0.1/1.3	0.0/0.2	0.0/0.1	0.0/0.0	0.18	0.33	0.25
11	0.54	0.75	0.3/0.5	0.1/0.4	0.1/0.4	0.0/0.0	0.0/0.0	0.0/0.0	0.18	0.33	0.25
12	0.54	0.75	0.1/1.8	0.0/1.1	1.0/2.6	0.0/0.3	0.0/0.3	0.0/0.4	0.18	0.33	0.25
13	0.54	0.75	0.2/0.8	0.4/0.6	0.0/0.3	0.0/0.2	0.0/0.2	0.0/0.0	0.18	0.33	0.25
14	0.54	0.75	0.8/2.1	0.3/0.8	0.9/2.2	0.0/0.4	0.0/0.4	0.0/0.2	0.18	0.33	0.25
15	0.54	0.75	0.3/1.0	0.1/0.5	0.1/0.4	0.0/0.0	0.0/0.0	0.0/0.0	0.18	0.33	0.25
16	0.54	0.75	0.7/1.4	0.2/0.6	0.3/0.8	0.0/0.1	0.0/0.2	0.0/0.0	0.18	0.33	0.25
17	0.54	0.75	0.6/1.3	0.2/1.0	0.4/1.9	0.0/0.3	0.0/0.2	0.0/0.1	0.18	0.33	0.25
18	0.54	0.75	0.2/0.7	0.1/0.6	0.1/0.9	0.0/0.0	0.0/0.0	0.0/0.1	0.18	0.33	0.25
19	0.54	0.75	0.6/1.4	0.2/0.8	0.3/0.9	0.0/0.3	0.0/0.3	0.0/0.1	0.18	0.33	0.25
20	0.54	0.75	0.2/0.5	0.1/0.5	0.1/0.4	0.0/0.0	0.0/0.0	0.0/0.0	0.18	0.33	0.25
21	0.54	0.75	0.2/0.8	0.2/0.9	0.2/1.0	0.0/0.0	0.0/0.0	0.0/0.1	0.18	0.33	0.25
22	0.54	0.75	0.3/1.0	0.1/0.8	0.3/0.9	0.0/0.1	0.0/0.0	0.0/0.1	0.18	0.33	0.25

**Table 4:** The uncertanity of segmentation error, deformation error, correlation error and prediction error and targeting error for each patient.

Patient	*U*_*S*_	*U*_*D*_	*U*_*C*_ (mm)			*U*_*P*_ (mm)			*U*_*T*_ (mm)		
number	(mm)	(mm)	S-I	L-R	A-P	S-I	L-R	A-P	S-I	L-R	A-P
1	1.46	1.5	0.9	0.9	0.5	0.0	0.0	0.0	0.49	0.86	0.64
2	1.46	1.5	0.6	1.0	0.5	0.0	0.0	0.0	0.49	0.86	0.64
3	1.46	1.5	0.7	1.1	1.1	0.0	0.0	0.0	0.49	0.86	0.64
4	1.46	1.5	0.8	0.9	0.9	0.0	0.0	0.0	0.49	0.86	0.64
5	1.46	2.5	0.8	0.4	0.4	0.0	0.0	0.0	0.49	0.86	0.64
6	1.46	2.5	0.8	0.4	1.0	0.0	0.0	0.0	0.49	0.86	0.64
7	1.46	1.5	0.5	0.4	0.2	0.0	0.0	0.0	0.49	0.86	0.64
8	1.46	1.5	1.5	0.3	0.2	0.0	0.0	0.0	0.49	0.86	0.64
9	1.46	1.5	0.2	0.2	0.2	0.0	0.0	0.0	0.49	0.86	0.64
10	1.46	1.5	0.8	0.2	0.2	0.0	0.0	0.0	0.49	0.86	0.64
11	1.46	1.5	0.3	0.2	0.2	0.0	0.0	0.0	0.49	0.86	0.64
12	1.46	1.5	1.5	0.2	2.9	0.0	0.0	0.0	0.49	0.86	0.64
13	1.46	1.5	0.8	1.3	0.2	0.0	0.0	0.0	0.49	0.86	0.64
14	1.46	1.5	3.1	0.7	3.0	0.0	0.0	0.0	0.49	0.86	0.64
15	1.46	1.5	0.9	0.5	0.5	0.0	0.0	0.0	0.49	0.86	0.64
16	1.46	1.5	2.6	0.6	1.2	0.0	0.0	0.0	0.49	0.86	0.64
17	1.46	1.5	1.7	0.8	2.1	0.0	0.0	0.0	0.49	0.86	0.64
18	1.46	1.5	0.5	0.3	0.3	0.0	0.0	0.0	0.49	0.86	0.64
19	1.46	1.5	2.0	0.5	1.0	0.0	0.0	0.0	0.49	0.86	0.64
20	1.46	1.5	0.6	0.4	0.4	0.0	0.0	0.0	0.49	0.86	0.64
21	1.46	1.5	0.6	0.7	1.1	0.0	0.0	0.0	0.49	0.86	0.64
22	1.46	1.5	0.8	0.3	0.7	0.0	0.0	0.0	0.49	0.86	0.64

Step 5: The targeting error calculated by the E2E tests was −0.13 ± 0.18 mm, 0.20 ± 0.33 mm, and −0.14 ± 0.25 mm for the S-I, L-R, and A-P directions, respectively, as shown in Table 3. The SD of the targeting error (∑_*T*_) was 0.18 mm, 0.33mm, and 0.25 mm for S-I, L-R, and A-P directions, respectively, as shown in Table 3. The uncertainty of the targeting error (${U}_{T}$) was 0.49 mm, 0.86 mm, and 0.64 mm for S-I, L-R, and A-P directions, respectively, as shown in Table 4.

### Individual margin

The values of the segmentation, deformation, correlation, prediction, and targeting errors are evaluated and shown in Table 3. The individual margin calculated using the formulation by van Herk *et al*.^26^ are shown in Table 5. These results represent an estimation of the margin to determine the position of 95% of the CTV points with a 90% confidence level during treatment. The uncertainty values of the segmentation, deformation, correlation, prediction, and targeting errors are evaluated and shown in Table 4. And the individual margin estimated by uncertainty method is also shown in Table 5. These results represent an estimation of the uncertainty to determine the position of 95% of the CTV points with a 95% confidence level during treatment. Based on these two results, we consider that: 1. A 3 mm global margin would provide 72.7% coverage in the S-I direction, 90.9% coverage in the L-R directions and 81.8% coverage in the A-P direction. 2. A 4 mm global margin would be able to provide a 95% coverage in the S-I direction and 100% coverage in the L-R and A-P directions. 3. A 5 mm global margin would be able to provide 100% coverage in all three directions.

**Table 5:** The margin determined from the segmentation, deformation, correlation, prediction and targeting errors using the validated formulation (M) and the uncertainty estimation method (U).

Patient number	M_S-I_ (mm)	M_L-R_ (mm)	M_A-P_ (mm)	U_S-I_ (mm)	U_L-R_ (mm)	U_A-P_ (mm)
1	2.6	2.7	2.5	2.4	2.5	2.3
2	2.5	2.8	2.6	2.3	2.5	2.3
3	2.7	2.9	2.8	2.3	2.6	2.5
4	2.6	2.7	2.7	2.3	2.5	2.4
5	3.6	3.6	3.5	3.1	3.1	3.0
6	3.6	3.6	3.8	3.1	3.1	3.2
7	2.5	2.5	2.5	2.3	2.3	2.2
8	4.6	2.6	2.5	2.7	2.3	2.2
9	2.5	2.5	2.5	2.2	2.3	2.2
10	3.2	2.8	2.6	2.3	2.3	2.2
11	2.5	2.5	2.5	2.2	2.3	2.2
12	2.6	2.6	3.9	2.7	2.3	3.7
13	2.5	2.7	2.4	2.3	2.7	2.2
14	3.4	2.7	3.6	3.8	2.4	3.8
15	2.6	2.5	2.5	2.4	2.4	2.3
16	3.1	2.6	2.6	3.4	2.4	2.5
17	3.0	2.6	2.9	2.8	2.5	3.1
18	2.5	2.5	2.5	2.3	2.3	2.3
19	3.0	2.6	2.6	3.0	2.4	2.5
20	2.5	2.5	2.5	2.3	2.3	2.3
21	2.5	2.6	2.6	2.3	2.4	2.5
22	2.6	2.6	2.6	2.3	2.3	2.3

### Statistics analysis

**Figure 3:**
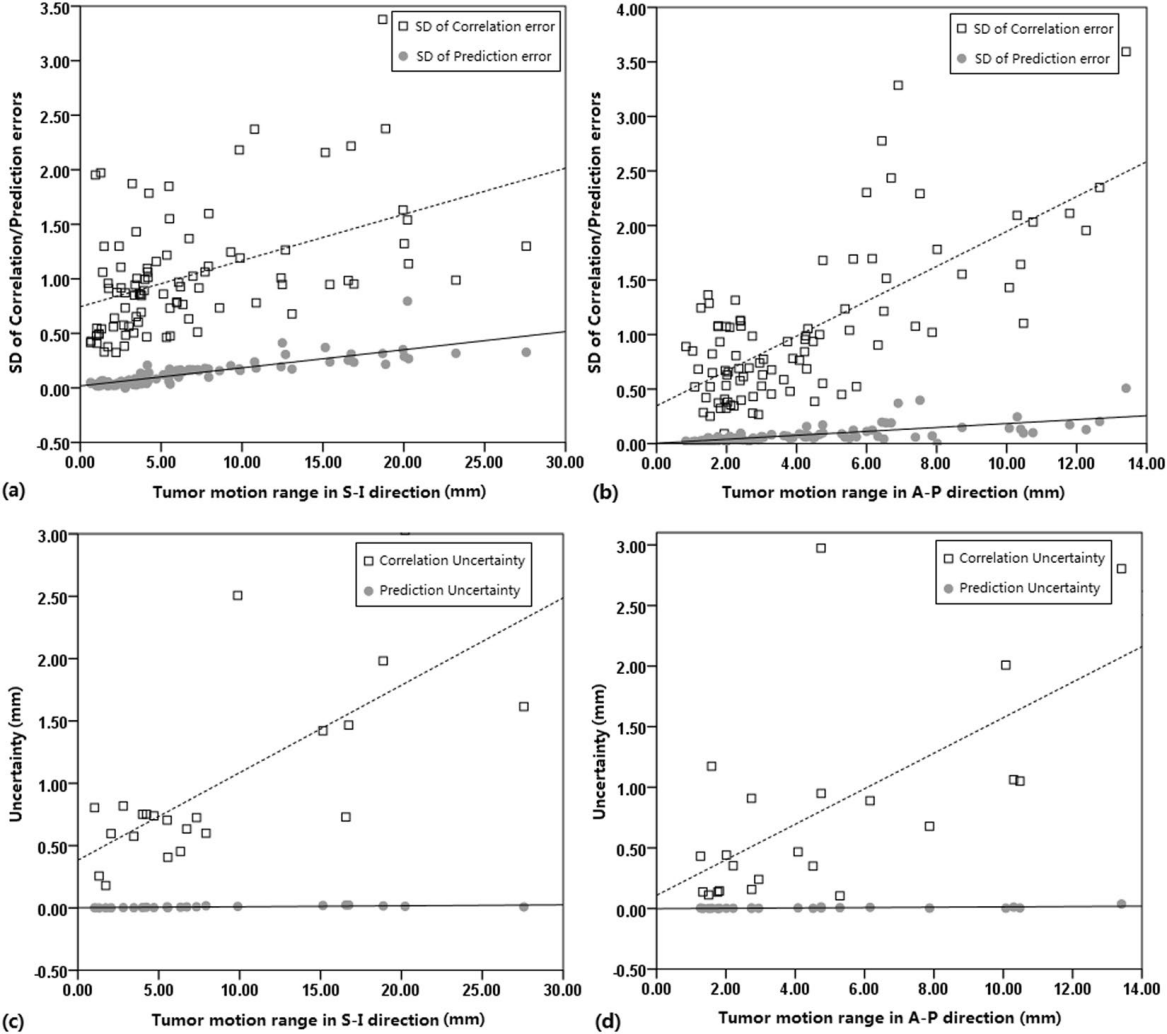
The correlation model lines of SD values of prediction or correlation errors and the tumor motion ranges in S-I and A-P directions, respectively.

The SD values of correlation errors and tumor motion ranges were significantly correlated in the S-I (R = 0.464, P < 0.001) and A-P (R = 0.712, P < 0.001) directions. The SD values of prediction errors and tumor motion ranges were significantly correlated in the S-I (R = 0.836, P < 0.001), L-R (R = 0.837, P < 0.001) and A-P (R = 0.673, P < 0.001) directions. The uncertainty of correlation errors and tumor motion ranges were significantly correlated in the S-I (R = 0.704, P < 0.001) and A-P (R = 0.642, P = 0.001) directions. The uncertainty of prediction errors and tumor motion ranges were significantly correlated in the S-I (R = 0.711, P < 0.001), L-R (R = 0.824, P < 0.001) and A-P (R = 0.632, P = 0.002) directions. The distributions of SD/uncertainty of correlation/prediction errors in the S-I and A-P directions and correlation model lines are shown in Fig. 3. There were no differences in margin and uncertainty of XLTS between the lesions located in upper/middle and lower lobe and tumor sizes. The uncertainty of XLTS of peripheral lesions were found higher than those of central lesions in S-I direction (P = 0.047) and A-P direction (P = 0.028). The uncertainty of XLTS of larger motion lesions were found significantly higher than those of smaller motion lesions in S-I direction (P = 0.004). The margin and uncertainty of XLTS of larger motion lesions were found significantly higher than those of smaller motion lesions in A-P direction (P = 0.022, P = 0.012).

## Discussion

Fiducial placement complications such as pneumothorax, pulmonary hemorrhage, systemic toxicity of local anesthetic drugs, and the risk of the inserted fiducials migrate or drop out, happened occasionally^9–11^. Obviously, the non-invasive XLTS is a superior tracking method; however, only two studies^8, 21^ have investigated the targeting errors of the CyberKnife XLTS, and there was no report about the margin of XLTS based on clinical data. This study has shown that the residual error was small when using the XLTS in clinical practice, and a 4 mm global margin could provide a 100% coverage in the L-R and A-P directions, and a 95% coverage in the S-I direction, with the individual margin ranging between 2.2 mm and 4.6 mm.

There are certain characteristics of the hypo-fractionated SBRT using the CyberKnife: 1) small target size; 2) small number of treatment fractions; 3) dose commonly prescribed at the sharp dose gradient position (65–85% of the maximum dose); 4) heterogeneous dose gradient along each spatial dimension; 5) intra-fraction variation pattern could be non-Gaussian, and heterogeneous in spatial dimensions. These characteristics represent that CyberKnife SBRT is difficulty for the margin design. However, other authors have applied the van Herk formalism to CyberKnife Synchrony data^17, 20^. Due to the large number of data points per treatment fraction, it was believed that equations (7), (8), and (9) properly represent the standard deviation of the random and systematic error, respectively. Descovich *et al*. performed a population margin analysis based on the van Herk formalism reported the ITV-to-PTV margin of lung tumors in CyberKnife treatments (FTTS or XLTS) were 6.81 mm in S-I direction, 4.42 mm in L-R direction and 4.67 mm in A-P direction^20^. Recently, there was a new method to calculate the tumor margin of CyberKnife FTTS treatment by the uncertainty analysis. Floriano *et al*. retrospectively analyzed the main sources of uncertainty in CyberKnife FTTS process patient by patient, and estimated their margin individually. They found a 5 mm margin is generally a safe geometric margin. Our results obtained using these two methodologies are compatible with the results of the abovementioned two authors. The margin calculated using the van Herk formalism were slightly larger than the margin estimated with uncertainty analysis. This small discrepancy may come from the differences of the two margin design methods. The van Herk formalism is a dosimetric margin design method, and it requires a larger statistical margin to cover the target during the whole treatment fractions. However, the uncertainty estimation method is based on the geometrical considerations only. And the designed margin using the uncertainty estimation method is also smaller comparing to the ones using the van Herk formalism in abovementioned two studies^14, 20^.

Because the segmentation error depends on tumor density, soft-tissue contrast, and the segmentation ability of X-ray images, it is difficult to be distinguished and observed during the treatment. We have to derive it from prior study. But the patient-specific lung phantoms could not represent the whole human body, for example, vertebral structures were not included. Thus this segmentation was likely easier than the actual situation where tumor itself could be obscured by vertebral structures. In addition, the phantom was moving only in the S-I direction, it could not represent the respiration trajectory in three directions. Due to the limitation of the phantom study, we chose the largest segmentation error 0.38 ± 0.54 mm reported in the study of Jung *et al*.^21^ to present the segmentation error in all three directions, but sometimes the segmentation error may still be underestimated. The tumor deformations correlated with the surrounding tissues have been studied previously^24, 25^. Lu *et al*. analyzed the CTV internal deformation of 12 lung patients for the fiducial migration and CTV coverage^25^. They found the prescription dose coverage over the CTV at the end-inspiration phase reduced to 90.2 ± 4.6% when the PTV had no margin to the CTV, but the coverage remains at >95% when the PTV had a 3 mm margin from the CTV^25^. This finding indicates that a 3 mm global margin is enough to cover the CTV internal deformation error. Smith *et al*. analyzed the deformation error in lung tumors and plot the deformation error as a function of the distance from the tumor centroid (Figure 5 in Smith *et al*.^24^). It shows that the motion deformation error increases as the distance from the tumor centroid increases. According to the abovementioned literatures, we set the motion range of the tracked target’s CoM as an input parameter for the deformation error value selection.

We found the uncertainties of XLTS of larger motion lesions were significantly higher than those of smaller motion lesions in S-I and A-P directions. The uncertainties of XLTS of peripheral lesions were found higher than those of central lesions in S-I and A-P directions. These results show that the tumor locations and motion ranges are the important factors that impact the tracking accuracy. We also found strong correlations between tumor motion ranges with the correlation/prediction errors, suggesting that the tumor motion ranges and regular respirations could lower the tracking accuracy. If patients could keep breathing regularly, they could gain a much more accurate real-time tracking treatment. Moreover, we found the correlation error was much higher than the prediction error. This is partially because of the correlation error contains the model error of motion trajectory (linear model, curvilinear model, dual-curvilinear model).

Our study has several limitations. First, the insufficient sample size has limited the generalizability of our results. Especially the lack of tumors located in the lower lobes included in our analysis may lead the margin coverage not enough for the larger motion range tumors. Second, the number of data included in this study is relatively small. When analyzing the correlation error, few data points of each fraction were available (<50 per fraction), due to the control of X-rays exposure of patients in the whole treatment. Third, the segmentation error and deformation error are estimated and calculated based on the prior studies. Since the prior studies may exist research bias, or be ideal and empirical, these two errors may have the risk of been underestimated sometimes.

This study has shown that in our center the total tracking error of the XLTS analyzed from the log files of clinical cases is less than 4 mm in all three directions. Our method of obtaining patient specific-margin would be to use a dry-run simulation treatment prior to formal treatment. Following the simulation treatment, the log files could be analyzed to determine margin specific to that patient. This study provides a reference method to the evaluation and selection of PTV margin for treatment with the CyberKnife XLTS.
